# Effectiveness of Acupotomy Combined with Epidural Steroid Injection for Lumbosacral Radiculopathy: A Randomized Controlled Pragmatic Pilot Study

**DOI:** 10.3390/medicina60010175

**Published:** 2024-01-19

**Authors:** Jin-Hyun Lee, Sang-Hyun Lee, Hae Sun Suh, Man-Suk Hwang, Semin Jang, Sooil Choi, Young-Soo Lim, Sang Hyun Byun, Sang-Hoon Yoon, Sukhee Park, Tae-Yong Park

**Affiliations:** 1 Institute for Integrative Medicine, International St. Mary’s Hospital, Catholic Kwandong University, Incheon 22711, Republic of Korea; doolyjinhyun@empal.com; 2 Department of Korean Medicine, Graduate School, Pusan National University, Yangsan 50612, Republic of Korea; leesh6498@gmail.com; 3 College of Pharmacy, Kyung Hee University, Seoul 02453, Republic of Korea; 4 Department of Regulatory Science, Graduate School, Kyung Hee University, Seoul 02453, Republic of Korea; 5 Institute of Regulatory Innovation through Science, Kyung Hee University, Seoul 02453, Republic of Korea; 6 Department of Korean Medicine Rehabilitation, Spine and Joint Center, Pusan National University Korean Medicine Hospital, Yangsan 50612, Republic of Korea; 7 Third Division of Clinical Medicine, School of Korean Medicine, Pusan National University, Yangsan 50612, Republic of Korea; 8 Department of Anesthesiology and Pain Medicine, International St. Mary’s Hospital, Catholic Kwandong University, Incheon 22711, Republic of Korea; swiri31@naver.com (S.C.);; 9 SAGAJEONG Pain and Korean Medicine Clinic, Seoul 02238, Republic of Korea; 10 Department of Applied Korean Medicine, Graduate School, Kyung Hee University, Seoul 02453, Republic of Korea

**Keywords:** acupotomy, epidural steroid injection, integrative medicine, pilot projects, radiculopathy, randomized controlled trial

## Abstract

*Background and Objectives*: This pilot study aimed to evaluate the clinical effectiveness, cost-effectiveness, and safety of acupotomy combined with epidural steroid injection (ESI) in lumbosacral radiculopathy and examine its feasibility for the main study. *Materials and Methods*: This randomized, controlled, two-arm, parallel, assessor-blinded, pragmatic study included 50 patients with severe lumbosacral radiculopathy who had insufficient improvement after an ESI. Patients were randomized (1:1 ratio) into a combined treatment (acupotomy + ESI, experimental) and an ESI single treatment (control) group. Both groups underwent a total of two ESIs once every 2 weeks; the experimental group received eight additional acupotomy treatments twice a week for 4 weeks. Types of ESI included interlaminar, transforaminal, and caudal approaches. Drugs used in ESI comprised a 5–10 mL mixture of dexamethasone sodium phosphate (2.5 mg), mepivacaine (0.3%), and hyaluronidase (1500 IU). The primary outcome was the difference in changes from baseline in the Oswestry Disability Index (ODI) scores between the groups at weeks 4 and 8. The incremental cost-utility ratio (ICUR) was calculated to evaluate the cost-effectiveness between the groups. Adverse events (AEs) were assessed at all visits. *Results*: Mean ODI scores for the experimental and control groups were −9.44 (95% confidence interval [CI]: −12.71, −6.17) and −2.16 (95% CI: −5.01, 0.69) at week 4, and −9.04 (95% CI: −12.09, −5.99) and −4.76 (95% CI: −7.68, −1.84) at week 8, respectively. The difference in ODI score changes was significant between the groups at week 4 (*p* = 0.0021). The ICUR of the experimental group versus the control group was as economical as 18,267,754 won/quality-adjusted life years. No serious AEs were observed. *Conclusions*: These results demonstrate the potential clinical effectiveness and cost-effectiveness of acupotomy combined with ESI for lumbosacral radiculopathy and its feasibility for a full-scale study. Larger, long-term follow-up clinical trials are needed to confirm these findings.

## 1. Introduction

Lumbosacral radiculopathy, which causes lumbar and radiating pain in the lower extremities by compressing the lumbar nerve root, encompasses lumbosacral spinal stenosis, intervertebral disc disorder, spondylosis, and neural canal stenosis [1]. The symptoms of lumbosacral radiculopathy are caused by a combination of biomechanical and biochemical factors [2]. Biomechanically, continuous nerve root compression due to structural problems disrupts the microvascular flow, which supplies blood to the nerves, and results in the activation of C fibers, which induces neuropathic pain [3]. Biochemically, pathological substances, such as interleukin-6 and tumor necrosis factor, are released into the proximal regions of the nerves through the phospholipase A2 pathway, potentially leading to neural damage [4]. Accordingly, related symptoms of lumbosacral radiculopathy, including motor degradation and radiating pain induced by neural damage, can deteriorate the quality of life and increase economic burden [5,6].

Among the various treatments used to manage this disease, epidural steroid injection (ESI), a nonsurgical, minimally invasive treatment, is a preferred treatment owing to its immediate effect [7]. The goal of ESI is to remove the pathological chemical substances induced by lumbosacral radiculopathy through the injection of steroids or anesthetics around the nerve root or in the epidural region [8]. However, long-term effects lasting more than 12 months are rare, and there is limited scope for the repetition of ESIs because of the side effects of steroids [9,10,11].

Recently, studies on acupotomy for lumbosacral radiculopathy have been conducted in South Korea and China [12,13,14]. Notably, Kwon et al. and Ye et al. conducted a systematic review on acupotomy for spinal stenosis and lumbar disc herniation [15,16], respectively, and suggested that acupotomy can be an effective treatment for lumbosacral radiculopathy. Acupotomy, a combination of acupuncture and microinvasive surgery, is excellent at resolving the mechanical compression force by cutting taut nodes and muscle cords surrounding the spine [17]. However, acupotomy may be insufficient to manage the inflammation in lumbosacral radiculopathy.

Hyeopcheok (EX-B2) acupoints, located in the L1–5 spinal erector muscle, are commonly used to treat lumbosacral radiculopathy [18,19]. With the theoretical effect of regulating the Governor Vessel and Bladder meridian simultaneously and communicating Yang qi throughout the body, the clinical effectiveness of deep needling at EX-B2 acupoints in lumbosacral radiculopathy has been proven in many studies [20,21]. Notably, Wang et al. compared an EX-B2 acupoint deep needling group with an EX-B2 acupoint ordinary needling group and demonstrated the superiority of deep needling over ordinary needling in lumbosacral radiculopathy [22].

This study aimed to control the pathological chemical substances and relieve mechanical soft tissue compression induced by lumbosacral radiculopathy with the following research hypothesis [23]: the combination of ESI, which has a chemical effect, and acupotomy, which has a mechanical effect, would create a synergy, resulting in better clinical outcomes. This pilot study aimed to evaluate the clinical effectiveness, cost-effectiveness, and safety of deeply inserted acupotomy to EX-B2 acupoints (DAH) combined with ESI for lumbosacral radiculopathy and to examine its feasibility before performing a full-scale main study.

## 2. Materials and Methods

### 2.1. Design

The protocol of this study was published in March 2022 [23]. This randomized, controlled, two-arm, parallel, assessor-blinded, pragmatic pilot study was conducted at Catholic Kwandong University, International St. Mary’s Hospital (CKUH), Incheon, Republic of Korea. This study design was approved by the institutional review board of CKUH (IS20OISE0085) and registered with the Clinical Research Information Service (www.cris.nih.go.kr; accessed on 14 December 2023) (trial registration number: KCT0006158, Registered on May 11 2021).

### 2.2. Participants

#### 2.2.1. Inclusion Criteria

The inclusion criteria were as follows: (1) participants aged 18–85 years; (2) patients diagnosed with spondylosis with lumbosacral intervertebral disc disorder (injury), spinal stenosis, or radiculopathy through imaging examinations (magnetic resonance imaging [MRI] or computed tomography [CT]) within 6 months prior to participation in this clinical trial (patients with suspected lumbosacral radiculopathy without imaging results underwent an MRI or CT scan on the day of screening for confirmation); (3) patients with symptoms related to lumbosacral radiculopathy (lumbosacral radiculopathy or lumbosacral neuropathy), such as radiating pain, hypotonia, and paresthesia in the lower extremities, or diagnosed with lumbosacral neuropathy through physical examination; (4) patients treated with one ESI for lumbosacral radiculopathy within 2 weeks before the start of the clinical trial and with <50% subjective improvement in pain after the procedure or pain rating index 5 or higher on the numerical rating scale (NRS); (5) patients who were able to read, understand, and answer the questionnaire; and (6) patients who voluntarily agreed and provided written informed consent for the procedure and follow-up monitoring.

#### 2.2.2. Exclusion Criteria

The following patients were excluded from this study: (1) those with a history of spinal surgery related to multiple cannulated screws, or spondylodesis in the lumbosacral region within 6 months prior to participation in the clinical trial, or who underwent spinal surgery in the past and still felt pain afterward; (2) those for whom surgical procedures were indicated owing to cauda equina syndrome or motor paralysis and neurological symptoms that may limit recovery by conservative treatment, as determined by the researchers; (3) those undergoing active treatment with drugs such as strong opioids for pain control; (4) those who had undergone acupotomy treatment within 2 weeks before the start of baseline screening for the clinical trial (patients were allowed to participate in this clinical trial if the researchers deemed them to have new neuropathic symptoms that were different from the existing pain, despite being treated in other centers within the relevant period); (5) those who had previously undergone ESI followed by side effects or related hypersensitivity reactions; (6) those with needle irritability, metal allergies, severe atopic dermatitis, keloid skin, and other dermal hypersensitivities; (7) those with hemophilia; (8) those taking drugs, such as anticoagulants, antiplatelets, and aspirin, that may cause hemostasis disorders and unable to discontinue treatment during the clinical trial as deemed by the researcher; (9) those who had participated in another clinical trial within 30 days before this clinical trial screening and received a medicinal product for that clinical trial (including placebo); (10) those with a history of psychotic disorders, alcoholism, and drug addiction; (11) those currently pregnant and lactating and of childbearing potential who were unwilling to take contraceptives during the clinical trial; and (12) those for whom participation in the clinical trial was considered to be inappropriate by researchers owing to other reasons.

### 2.3. Randomization and Blinding

**Figure 1:**
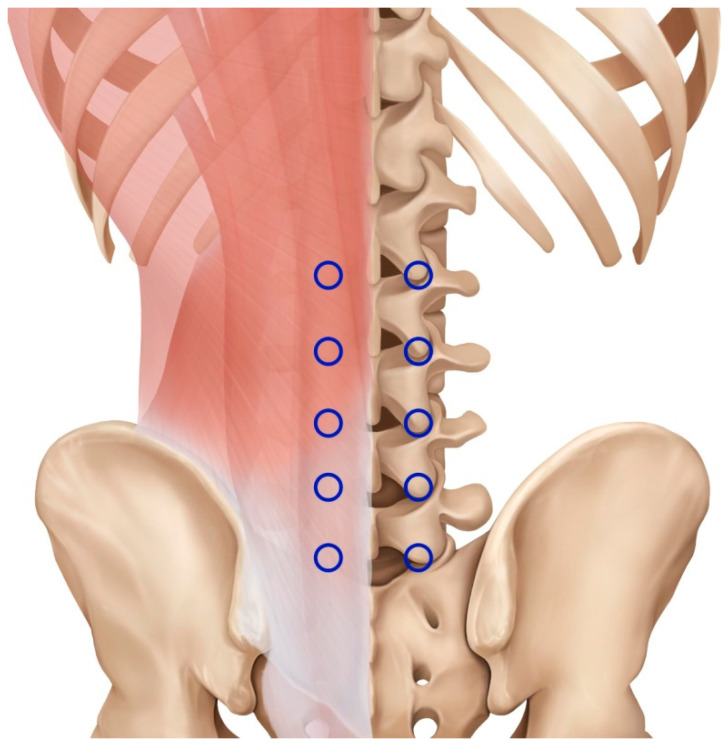
Location of the Hyeopcheok acupoint (EX-B2 L1-5). Each blue circle indicates the location of EX-B2 in the lumbar spine. EX-B2 is the international standard nomenclature for Hyeopcheok acupoints.

Fifty patients with lumbosacral radiculopathy who met the inclusion criteria were recruited and randomly assigned to the DAH (Figure 1) + ESI treatment group (experimental, n = 25) and ESI single-treatment group (control, n = 25) at a ratio of 1:1. A randomization table and envelopes were created by an independent professional statistician and an individual who was not involved in this study, respectively. After obtaining informed consent, screening numbers were assigned according to the order of the patients’ visits. The randomization envelopes were then distributed according to the order of assignment of the patients, who were assigned to one of the two groups. Blinding of the researchers and participants was not possible owing to the nature of the acupotomy. Thus, independent researchers who did not participate in the treatment evaluated the outcomes while maintaining blindness to prevent bias.

### 2.4. Interventions

Both groups underwent a total of two ESIs once every 2 weeks during the 4 weeks of treatment. The types of ESI included interlaminar, transforaminal, and caudal approaches. For safety, ESIs were performed using a C-arm (Siemens, München, Germany). The drugs used in ESI were a 5–10 mL mixture of dexamethasone sodium phosphate (2.5 mg), mepivacaine (0.3%), and hyaluronidase (1500 IU). All ESI procedures were performed by anesthesiologists who had at least 10 years of clinical experience. The treatment method, site, and dose could be freely performed based on the discretion of the person in charge of the procedure.

The experimental group additionally received a total of eight DAH treatments twice a week. Dongbang acupotomy (Dongbang Medical, Seongnam-si, Republic of Korea) with specifications of 0.5 × 50 or 80 mm, 0.75 × 50 or 80 mm, or Hansung Ahn’s small round needle (Hanson Precision Manufacture, Seoul, Republic of Korea) with specifications of 0.7 × 50 or 80 mm was used for DAH treatment. The treatment procedure was as follows: first, an ice pack was applied for less than 20 min to the skin of the lower back to reduce pain. Next, alcohol and povidone were applied to disinfect the treatment area. Subsequently, DAH treatment was performed on the disinfected treatment area under the guidance of ultrasound (GE Ultrasound Korea, Seongnam-si, Republic of Korea). Ultrasound was used to track the tip of the acupotomy to avoid nerves and blood vessels for safety (Supplemental Video S1). When a response such as the de-qi sensation occurred, the needle was immediately removed. After the procedure, the treatment area was pressed for 3 min using sterile gauze to prevent hemostasis. All DAH treatment procedures were performed by Korean medicine doctors who had at least 10 years of clinical experience. DAH treatment was performed on EX-B2 acupoints of the lumbar level related to the patient’s clinical symptoms, such as lumbar and radiating pain, and based on imaging exams (MRI or CT) and physical examination. Additionally, acupoints in the first line of the Bladder meridian (1.5 cun lateral to the spine) or Ashi points around the affected area were selected at the operator’s discretion. To maximize the mechanical stimulating effect of acupotomy, we inserted the needle more deeply in EX-B2 acupoints than during usual acupuncture or acupotomy [24,25]. EX-B2 acupoints were used to directly provide physical stimulation to the affected area. Considering that the depth of the L1-5 transverse processes is 45–58 mm, the needle depth was set to within 50–60 mm at the time of perpendicular insertion (Supplemental Table S1) [26].

### 2.5. Outcome Measures

The primary outcome was the difference in changes from baseline in the Korean version of the Oswestry Disability Index (ODI) between the groups at weeks 4 (immediately after treatment) and 8 (4 weeks after the end of treatment) [27]. Section 4, the sex life questionnaire in the ODI assessment, was not assessed considering the cultural context of the general Korean population. The secondary outcomes were the differences in changes from baseline in the NRS of lumbar and lower limb pain [28], European Quality of Life 5 Dimensions (EQ-5D) [29], McGill Pain Questionnaire (MPQ) [30], and the Korean version of the Roland–Morris Disability Questionnaire (RMDQ) [31] between the groups at weeks 4 and 8 (Supplemental Table S2).

We assessed additional outcomes, including the proportion of patients who underwent additional treatments, rate of early termination, usage of emergency medications, and ratio of treatment responder/nonresponder assessment (ODI and NRS) [32], for further explorative effectiveness evaluations (Supplemental Table S2). The additional procedure rate was calculated as the proportion of patients who underwent additional ESIs or surgeries among those who completed the entire study. The early termination rate was calculated as the proportion of patients who terminated this study prematurely among the total number of patients who completed the study.

Adverse events (AEs) were assessed at all visits after randomization, including physical examination, vital signs, and subjective and objective symptoms. In addition, safety was assessed using the Patient-Reported Outcomes version of the Common Terminology Criteria for Adverse Events (PRO-CTCAE), in which patients self-reported their overall health condition (Supplemental Table S2) [33].

For the evaluation of cost-effectiveness, the Korean-specific score system (Korean tariff) based on the study by Jo et al. was applied to utility scores [34], which measured the health status of each group with the EQ-5D (preference-based tool), by which the quality-adjusted life years (QALYs) of the health condition were calculated. In this study, a societal perspective was employed, and cost items were collected, including direct medical, nonmedical, and productivity loss costs via the Institute for Medical Technology Assessment productivity cost questionnaire at baseline and at weeks 2, 4, and 8. The incremental cost-utility ratio (ICUR) was calculated to evaluate cost-effectiveness based on the difference in cost and health-related QALYs between the two groups. Specific items for each cost are described in the existing published protocol [23].

### 2.6. Sample Size Calculation

This was a pilot study assessing the feasibility of the study design for exploring the effectiveness and safety of DAH + ESI treatment. Therefore, it was necessary to proceed with the minimum number of participants to meet the purpose of this study. A previously reported study with a design comparable to that of this study was used as a reference [13]. In the aforementioned study, acupotomy (n = 20) and acupuncture (n = 20) were compared in 40 patients with lumbar disc herniation. Based on that study, the present study aimed to recruit a total of 50 participants (25 per group), considering a dropout rate of 20%.

### 2.7. Statistical Analysis

All analyses were based on the intention-to-treat principle, and missing data were imputed with the baseline observation carried forward analysis imputation method. The changes in the primary and secondary outcomes were compared using ANCOVA, with the baseline scores as covariates and the group as the fixed factor. In addition, the improvement ratio between the groups was analyzed using the Chi-square or Fisher’s exact tests. The treatment safety assessment included all patients who had been treated at least once. The Chi-square or Fisher’s exact tests were used to determine whether there were any differences in the incidence ratio between the groups. This study was performed with a two-tailed test at a significance level of 5% (α = 0.05). All statistical analyses were performed using SAS version 9.4 (SAS Institute Inc., Cary, NC, USA).

## 3. Results

### 3.1. Participants

**Figure 2:**
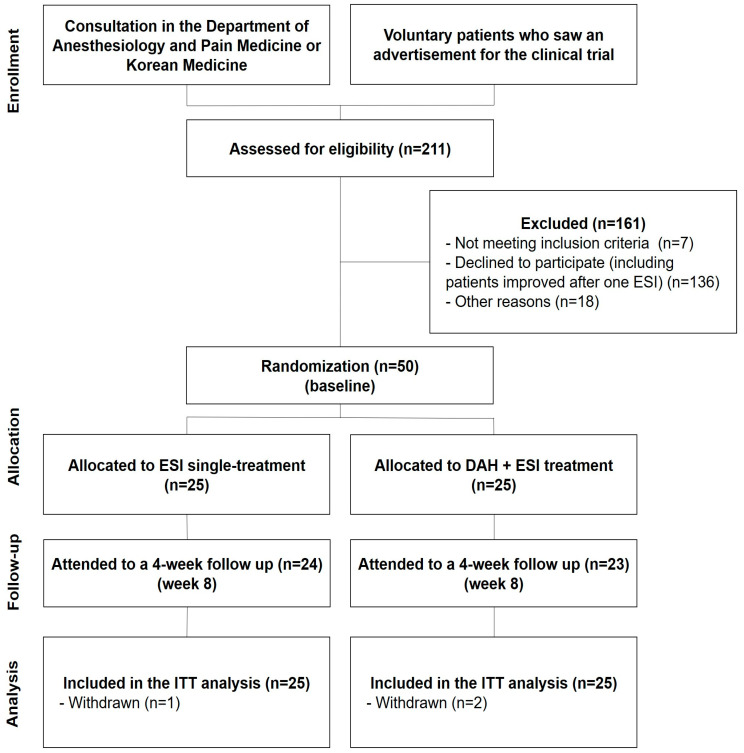
CONSORT flowchart of participant enrollment. Abbreviations: DAH, deeply inserted acupotomy to Hyeopcheok acupoints; ESI, epidural steroid injection; ITT, intention-to-treat.

A flowchart of patient recruitment and the overall study is presented in Figure 2. In total, 211 patients with lumbosacral radiculopathy were treated with one ESI between 2 March 2021, and 15 April 2022. After one ESI, patients were screened according to the inclusion and exclusion criteria. Consequently, 161 patients were excluded, and 50 patients were enrolled. During this study, two participants in the experimental group withdrew their consent and stopped participating at visits 3 and 5, respectively, and one participant in the control group withdrew their consent and stopped participating at visit 5 (Figure 2).

### 3.2. Baseline Characteristics

The summary data for each demographic characteristic for the experimental and control groups and the results of the group tests are shown in Table 1. Statistical differences in age, sex, height, weight, body mass index, and major factors were not observed between the groups.

### 3.3. Outcomes

#### 3.3.1. Primary Outcomes

**Figure 3:**
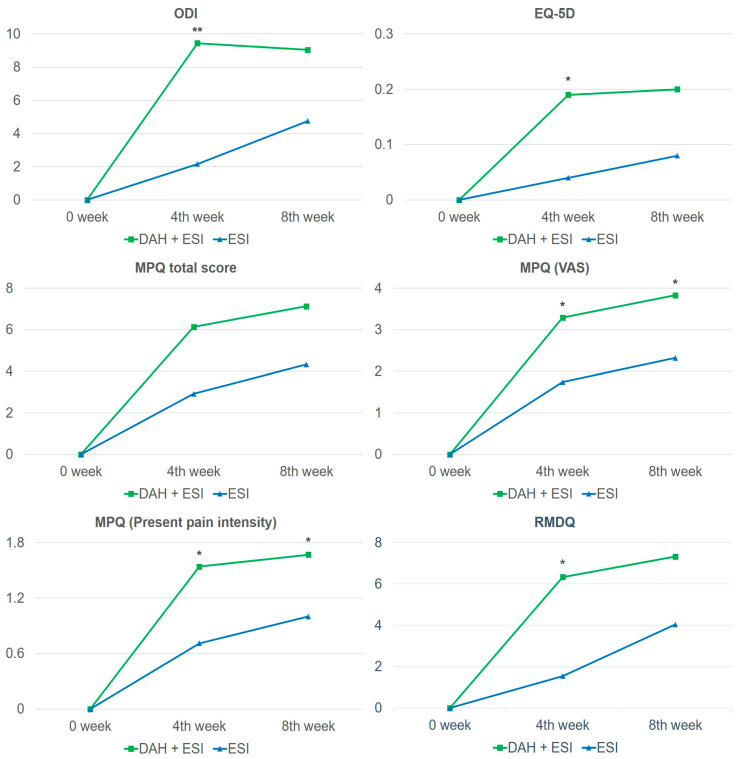
Line graph of changes from baseline between the experimental and control groups. Green, DAH + ESI treatment group; Blue, ESI single-treatment group. * *p* < 0.05, ** *p* < 0.01. Abbreviations: DAH, deeply inserted acupotomy to Hyeopcheok acupoints; EQ-5D, European Quality of Life 5 Dimensions; ESI, epidural steroid injection; MPQ, McGill Pain Questionnaire; ODI, Oswestry Disability Index; RMDQ, Roland–Morris Disability Questionnaire; VAS, visual analog scale.

The outcomes in both groups at baseline and weeks 4 and 8, and changes at weeks 4 and 8 relative to baseline, are shown in Table 2. The mean ODI scores at week 4 for the experimental and control groups decreased by 9.44 (95% confidence interval [CI]: −12.71, −6.17) and 2.16 (95% CI: −5.01, 0.69), respectively, compared to baseline, showing a treatment effect in both groups. Moreover, the two groups had statistically significant differences in the changes in ODI scores at week 4 (*p* = 0.0021). The mean ODI score at week 8 decreased by 9.04 (95% CI: −12.09, −5.99) and 4.76 (95% CI: −7.68, −1.84), respectively, compared to baseline, showing a treatment effect in both groups; however, no statistically significant difference was observed between the groups at week 8 (Table 2 and Figure 3).

#### 3.3.2. Secondary Outcomes

In examining the secondary outcomes, outcomes with statistically significant differences between the two groups at week 4 were EQ-5D (*p* = 0.0161), MPQ (visual analog scale [VAS]) (*p* = 0.0388), MPQ (present pain intensity) (*p* = 0.0172), and RMDQ (*p* = 0.0207). However, differences in the changes in EQ-5D and RMDQ scores between the groups were not significant at week 8. Meanwhile, differences in the changes in MPQ (VAS) (*p* = 0.0257) and MPQ (present pain intensity) (*p* = 0.0372) were also significant at week 8, and between-group differences in MPQ (sense), which were not statistically significant at week 4, were significant at week 8 (*p* = 0.0477) (Table 2 and Figure 3).

#### 3.3.3. Evaluation of Outcomes

Regarding the additional procedure rate, 47 patients completed the treatment, and four patients underwent additional procedures, with two each in the experimental and control groups (Supplemental Table S3). The additional procedures were ESI (one patient each in the experimental and control groups), medial branch block (one in the experimental group), and radiofrequency treatment (one in the control group). No differences were observed between the groups.

For the early termination rate, 47 patients completed the treatment, and no patients prematurely terminated the study; hence, the groups were not compared.

Rescue medications, including nonsteroidal anti-inflammatory analgesics, neuralgic treatments, digestive medicines, antidepressants, skeletal muscle relaxants, weak opioids, and anticonvulsants, were used by 20 patients in the control group and 15 patients in the experimental group. No between-group differences were observed (Supplemental Table S4).

The treatment responder/nonresponder group evaluation showed statistically significant between-group differences in the number of treatment responders/nonresponders as indicated by the ODI, while no statistically significant between-group differences were observed in the number of treatment responders/nonresponders as indicated by the NRS (Supplemental Table S3). However, the responder group showed a statistically significant reduction in lumbar and lower limb pain, as indicated by NRS scores (NRS score > 2.5).

#### 3.3.4. Cost-Effectiveness

From a societal perspective, the ICUR of DAH + ESI treatment versus ESI single-treatment was measured at −18,267,754 won/QALYs (Table 3). In particular, the cost of productivity loss measured at week 8 was the lowest (baseline: 291,764 won vs. week 8: 92,591 won) for the experimental group during the entire clinical trial period, while it was similar to the value at baseline for the control group (baseline: 283,120 won vs. week 8: 276,099 won).

#### 3.3.5. Safety and AEs

When comparing the number of participants with grade 4 or higher scores on the PRO-CTCAE survey questions, no between-group difference was observed (Supplemental Table S5). A generalized estimating equation analysis was not performed because no clinically significant changes (grade 4 or higher) were found in the results of the PRO-CTCAE surveys.

In total, eight patients experienced AEs, including three patients in the control group and five patients in the experimental group. Three patients in the control group experienced mild AEs of abdominal discomfort, pain in both legs, and knee-joint pain, and five patients in the experimental group experienced moderate AEs, including exacerbations of existing lumbar or lower limb pain (two patients), left shoulder pain due to a traffic accident, knee pain, and dizziness.

The results of clinical laboratory tests showed that no statistically significant differences were observed between the groups except in sodium (Na) levels. The control group had a significantly higher level of Na than the experimental group (*p* = 0.0375) (Supplemental Table S6).

## 4. Discussion

In various musculoskeletal pain disorders, the combined treatment of Western medicine and complementary medicine has often shown good results in both patient satisfaction and treatment effect [35]. In fact, Korea has a dualized health-care system of Western and Korean medicine, and patients with musculoskeletal pain actively receive treatments under both systems [36]. However, regarding lumbosacral radiculopathy, few studies have evaluated the synergistic effectiveness, cost-effectiveness, and safety of combined treatment with Western and Korean medicine, apart from the preference for integrative treatment in actual Korean clinical practice.

Since acupotomy can control biomechanical factors with its excellent physical stimulation effect and ESI can control biochemical factors by injecting steroids, we expected that combining the two interventions could synergize their advantages while minimizing their limitations. Based on the results of this trial, combining DAH during the implementation of ESI could be more effective in relieving pain and improving the function of the lower back and activities of daily living compared with ESI alone. Furthermore, combined DAH and ESI treatment appeared to be a dominant strategy compared with ESI single-treatment in terms of cost-effectiveness. Notably, the decrease in the cost of productivity loss in the combined treatment group showed the potential for a reduction in unnecessary costs and cost savings to society as a whole. Additionally, all eight AEs that occurred were not serious; thus, we were also able to ensure safety outcomes. According to a study conducted in China, acupotomy can cause mild AEs such as bruises, erythema, and needling pain to severe AEs such as syncope, nerve damage, and infection [37]. However, compared with acupotomy practiced in China, acupotomy practiced in Korea is smaller in size, less invasive, and therefore less risky. Yoon et al. evaluated 258 acupotomy treatments and observed low AE incidence (3.11% and 2.28% for systemic and local AEs, respectively) and no serious AEs [38]. In this study, there were five AEs in the experimental group; however, considering that acupotomy was inserted deeper than normal acupuncture and no serious AEs occurred, it was possible to ensure the safety of DAH.

The results indicate that combined DAH and ESI treatment can be clinically effective, cost-effective, and safe for patients with lumbosacral radiculopathy in real-world clinical practice. This study adopted a pragmatic trial approach. Rather than merely confirming the efficacy of a specific intervention, the authors tried to investigate the effectiveness that arises when combining treatment interventions widely utilized in actual clinical settings.

This study has the following strengths: First, a combined treatment of Western and Korean medicine was performed and showed a synergistic effect. Many clinical studies on lumbosacral radiculopathy used Western and Korean medicine interventions separately in practice or lacked a synergistic viewpoint regarding the advantages of both from an integrated medical perspective. The present study has a design similar to that utilized by Gao et al. and Gui et al., in that we compared the combined treatment of ESI and acupotomy with ESI single-treatment for lumbosacral radiculopathy [39,40]. However, this study can be considered systematically better than these previous studies in that we targeted patients with severe pain who had only a slight improvement in pain even after a session of ESI, used specific acupoints called EX-B2, performed cost-effectiveness evaluations, and applied ultrasound-guided acupotomy for safety. Second, the disadvantage of ESI being insufficient to improve the structural problems related to the spine was solved by acupotomy instead of invasive treatment. Moreover, effectiveness, cost-effectiveness, and safety outcomes were obtained. According to the results of a study that evaluated the cost-effectiveness of acupuncture [41], acupotomy, an acupuncture-like intervention, can also be expected to be cost-effective. However, there are no previous studies that evaluated the cost-effectiveness of acupotomy; hence, this is the first study to do so. In addition, we were able to obtain accurate data with a low patient dropout rate, and although eight patients experienced AEs, DAH + ESI treatment was found to be safe. Third, standard operating procedures (SOPs) were established through the Standardization Committee for DAH, according to how the researchers performed the treatments. The SOP education was conducted twice, and the procedure site was agreed upon between the clinical investigators. The established SOP allowed us to perform the same DAH procedure with the study participants, enabling us to obtain objective outcomes. Fourth, this study was funded by a national agency, and the quality of this study was secured through the rigorous management of the Contract Research Organization (CRO) and the development of electronic case report forms (e-CRFs). E-CRFs enabled the CRO to perform continuous monitoring and data management to further improve the quality of the study.

This study has some limitations. First, the 4-week follow-up period was relatively short; thus, the long-term effects of DAH could not be evaluated. In addition, a small number of participants were recruited from a single institution by calculating the sample size based on a similar study [13], owing to the absence of studies with the same design. Therefore, the full-scale study or other future studies should include more participants and use a longer follow-up period to evaluate the long-term effectiveness of combined DAH and ESI treatment.

## 5. Conclusions

This pilot study has demonstrated sufficient feasibility for the full-scale study. The chemical effect of ESI and the mechanical effect of acupotomy were synergistic, and the clinical effectiveness, cost-effectiveness, and safety of combined DAH and ESI treatment could be objectively investigated. If the full-scale study determines surgical morbidity through a long-term follow-up, the positive effects of the combined DAH and ESI treatment would be more clearly demonstrated. Based on the results of this pilot study, a randomized controlled trial with an extended scale and long-term follow-up is warranted to confirm the clinical effectiveness, cost-effectiveness, and safety of combined DAH and ESI treatment.

## Figures and Tables

**Table 1:** Demographic characteristics.

	ESI Single-Treatment Group	DAH + ESI Treatment Group	*p*-Value
Sample size (n)	25	25	
Age (years) ^a^	63.2 ± 10.7	60.8 ± 13.1	0.4749
Sex (n) ^b^			
Male	13 (52)	8 (32)	0.1520
Female	12 (48)	17 (68)	
Height (cm) ^a^	161.3 ± 8.6	159.4 ± 9.4	0.4573
Body weight (kg) ^a^	64.9 ± 12.1	62.9 ± 14.8	0.5995
BMI (kg/m^2^) ^a^	24.8 ± 3.8	24.4 ± 3.6	0.6902
SBP (mmHg) ^a^	128.9 ± 15.6	131.2 ± 10.0	0.5419
DBP (mmHg) ^a^	69.3 ± 14.8	74.9 ± 7.4	0.0996
Pathology (n) ^c^			
Spinal stenosis	11 (44)	6 (24)	0.4100
Intervertebral disc disorder	11 (44)	14 (56)	
Others	3 (12)	5 (20)	
Duration of pain (days)	255.0 (93.0, 949.0)	106.0 (57.0, 454.0)	0.2274
Type of ESI—first (n) ^c^			
Caudal block	4 (16)	5 (20)	1.0000
Interlaminar approach	21 (84)	20 (80)	
Type of ESI—second (n) ^c^			
Caudal block	6 (24)	5 (20.83)	0.7906
Interlaminar approach	19 (76)	19 (79.17)	

All values are presented as the mean ± standard deviation, median (Q1, Q3), or number (%). Analysis of variance is performed for all continuous variables, satisfying normality and equal variance. ^a^ Independent-samples *t*-test; ^b^ χ2 test; ^c^ Fisher’s exact test. Abbreviations: BMI, body mass index; DAH, deeply inserted acupotomy to Hyeopcheok acupoints; DBP, diastolic blood pressure; ESI, epidural steroid injection; SBP, systolic blood pressure.

**Table 2:** Comparison of outcomes and changes from baseline at each time point between the experimental and control groups.

	Outcomes			Changes from Baseline		
	ESI Single-Treatment Group (n = 25)	DAH + ESI Treatment Group (n = 25)	*p*-Value	ESI Single-Treatment Group (n = 25)	DAH + ESI Treatment Group (n = 25)	*p*-Value ^c^
ODI score						
Baseline	27.64 (25.61, 29.67)	29.24 (26.46, 32.02)	0.3418			
Week 4	25.48 (22.17, 28.79)	19.80 (16.62, 22.98)	0.0139 ^a^	−2.16 (−5.01, 0.69)	−9.44 (−12.71, −6.17)	0.0021 ^b^
Week 8	22.88 (19.66, 26.10)	20.20 (17.74, 22.66)	0.1791	−4.76 (−7.68, −1.84)	−9.04 (−12.09, −5.99)	0.0751
NRS (back pain)						
Baseline	6.08 (5.13, 7.03)	6.80 (5.71, 7.89)	0.3087			
Week 4	5.28 (4.09, 6.47)	4.16 (3.10, 5.22)	0.1549	−0.80 (−2.35, 0.75)	−2.64 (−3.85, −1.43)	0.1144
Week 8	5.12 (4.13, 6.11)	4.20 (3.22, 5.18)	0.1787	−0.96 (−2.08, 0.16)	−2.60 (−3.70, −1.50)	0.0673
NRS (lower limb pain)						
Baseline	7.84 (7.06, 8.62)	8.28 (7.74, 8.82)	0.3425			
Week 4	6.04 (5.05, 7.03)	5.04 (3.83, 6.25)	0.1928	−1.80 (−2.50, −1.10)	−3.24 (−4.60, −1.88)	0.0942
Week 8	5.48 (4.44, 6.52)	4.80 (3.85, 5.75)	0.3230	−2.36 (−3.19, −1.53)	−3.48 (−4.62, −2.34)	0.1776
EQ-5D						
Baseline	0.63 (0.57, 0.69)	0.58 (0.51, 0.65)	0.2695			
Week 4	0.67 (0.60, 0.74)	0.77 (0.70, 0.84)	0.0210 ^a^	0.04 (−0.04, 0.11)	0.19 (0.10, 0.28)	0.0161 ^a^
Week 8	0.71 (0.64, 0.78)	0.78 (0.72, 0.83)	0.0658	0.08 (0.02, 0.15)	0.20 (0.12, 0.28)	0.0511
MPQ total score						
Baseline	26.44 (23.92, 28.96)	27.12 (24.65, 29.59)	0.6922			
Week 4	22.96 (19.49, 26.43)	20.71 (18.23, 23.18)	0.2808	−2.92 (−5.44, −0.39)	−6.13 (−8.69, −3.56)	0.0910
Week 8	21.54 (18.97, 24.12)	19.71 (17.75, 21.67)	0.2476	−4.33 (−6.40, −2.27)	−7.13 (−9.34, −4.91)	0.0726
MPQ (sense)						
Baseline	19.04 (17.12, 20.96)	19.28 (17.62, 20.94)	0.8461			
Week 4	17.17 (14.58, 19.75)	15.38 (13.7, 17.05)	0.2352	−1.42 (−3.11, 0.28)	−3.71 (−5.26, −2.16)	0.0520
Week 8	16.33 (14.45, 18.22)	14.79 (13.36, 16.22)	0.1840	−2.25 (−3.86, −0.64)	−4.29 (−5.62, −2.97)	0.0477 ^a^
MPQ (emotion)						
Baseline	7.40 (6.40, 8.40)	7.84 (6.8, 8.88)	0.5308			
Week 4	5.79 (4.83, 6.76)	5.33 (4.39, 6.28)	0.4862	−1.50 (−2.64, −0.36)	−2.42 (−3.56, −1.28)	0.3354
Week 8	5.21 (4.40, 6.02)	4.92 (4.19, 5.64)	0.5808	−2.08 (−3.06, −1.11)	−2.83 (−3.89, −1.77)	0.3876
MPQ (VAS)						
Baseline	8.21 (7.55, 8.87)	8.40 (7.82, 8.98)	0.6541			
Week 4	6.21 (5.24, 7.17)	5.08 (4.13, 6.04)	0.0933	−1.74 (−2.62, −0.86)	−3.29 (−4.40, −2.18)	0.0388 ^a^
Week 8	5.48 (4.60, 6.35)	4.54 (3.75, 5.33)	0.1053	−2.32 (−3.06, −1.58)	−3.83 (−4.83, −2.83)	0.0257 ^a^
MPQ (present pain intensity)						
Baseline	3.88 (3.63, 4.13)	3.96 (3.61, 4.31)	0.7002			
Week 4	3.13 (2.65, 3.60)	2.46 (2.03, 2.89)	0.0360 ^a^	−0.71 (−1.13, −0.29)	−1.54 (−2.04, −1.04)	0.0172 ^a^
Week 8	2.83 (2.43, 3.24)	2.33 (2.01, 2.65)	0.0520	−1.00 (−1.41, −0.59)	−1.67 (−2.11, −1.22)	0.0372 ^a^
RMDQ						
Baseline	11.12 (9.03, 13.21)	12.24 (9.59, 14.89)	0.4963			
Week 4	9.04 (6.65, 11.43)	6.62 (3.42, 9.81)	0.2070	−1.54 (−4.05, 0.97)	−6.33 (−8.73, −3.93)	0.0207 ^a^
Week 8	6.74 (4.90, 8.57)	5.84 (3.39, 8.29)	0.5364	−4.04 (−6.44, −1.64)	−7.32 (−9.88, −4.76)	0.1939

All values are presented as the mean (95% confidence intervals). ^a^ *p* < 0.05, ^b^ *p* < 0.01. ^c^ Least squares mean difference and *p*-values were analyzed using ANCOVA with the baseline scores as covariates and the group as the fixed factor. Abbreviations: CI: confidence interval; DAH, deeply inserted acupotomy to Hyeopcheok acupoints; EQ-5D, European Quality of Life: 5 Dimensions; ESI, epidural steroid injection; MPQ, McGill Pain Questionnaire; NRS, Numeral Rating Scale; ODI, Oswestry Disability Index; RMDQ, Roland–Morris Disability Questionnaire; VAS, visual analog scale.

**Table 3:** ICUR of DAH + ESI treatment compared with ESI single-treatment from a societal perspective.

	ESI Single-Treatment Group	DAH + ESI Treatment Group	Increment
Cost (Won)	978,817	788,467	−190,350
QALYs	0.10271	0.11313	0.01042
ICUR (Won/QALYs)	−18,267,754		

Abbreviations: DAH, deeply inserted acupotomy to Hyeopcheok acupoints; ESI, epidural steroid injection; ICUR, incremental cost-utility ratio; QALYs, quality-adjusted life years.

## Data Availability

The data that supports the findings of this study are available from the corresponding author upon reasonable request.

## Supplementary Materials

The following supporting information can be downloaded at: https://www.mdpi.com/article/10.3390/medicina60010175/s1. Table S1: Checklist for Standards for Reporting Interventions in Clinical Trials of Acupuncture (STRICTA); Table S2: Outcomes observation schedule; Table S3: Comparison of the number of additional treatments and the number of responders/nonresponders in the experimental and control groups; Table S4: Comparison of rescue medication usage between the experimental and control groups; Table S5: Comparison of high-grade (score ≥ 4) PRO-CTCAE between the experimental and control groups; Table S6: Comparison of the clinical laboratory examination results between the experimental and control groups; Video S1: Ultrasound was used to track the tip of the acupotomy to avoid nerves and blood vessels for safety.
