# Supplementary material for: Effectiveness of Acupotomy Combined with Epidural Steroid Injection for Lumbosacral Radiculopathy: A Randomized Controlled Pragmatic Pilot Study
Source: Medicina (Kaunas). 2024 Jan 19;60(1):175. doi: 10.3390/medicina60010175 (PMC10818306; doi:10.3390/medicina60010175)
Supplement: Supplementary file 1 [file medicina-60-00175-s001.zip › Supplemental File.pdf]

Supplementary Materials

Table S1 Checklist for Standards for Reporting Interventions in Clinical Trials of Acupuncture (STRICTA)

Item	Detail	Explanation
**1. Acupuncture rationale**	1a) Style of acupuncture	Acupotomy<br>- For treating lumbosacral radiculopathy, the present study performs acupotomy on the areas determined by the Standardization Committee for deeply inserted acupotomy to Hyeopcheok (EX-B2) acupoints.
	1b) Reasoning for treatment provided, based on historical context, literature sources, and/or consensus methods, with references where appropriate	1) Preceding studies related to acupotomy<br>2) Consensus derived by the acupotomy standardization committee
	1c) Extent to which treatment was varied	1) EX-B2 at the level that can be the cause of lumbosacral radiculopathy – essential acupoint<br>2) Acupoints in the first line of the Bladder meridian or Ashi points around the affected area selected at the researcher's discretion: selectable acupoints
**2. Details of needling**	2a) Number of needle insertions per participant per session (mean	Determination of the proper number of needles at the

	and range where relevant)	researcher’s discretion
	2b) Names (or location if no standard name) of points used (uni/bilateral)	1) EX-B2<br>2) BL22, BL23, BL24, BL25, BL26<br>3) Ashi points around the affected area
	2c) Depth of insertion, based on a specified unit of measurement, or on a particular tissue level	1) Perpendicular insertion - 50–60 mm<br>2) Obliquely inserted - 70–80 mm
	2d) Response sought	1) Sensation such as de-qi sensation or SanMaJungChang<br>2) Induration reduction of the procedure site
	2e) Needle stimulation	Manual acupuncture
	2f) Needle retention time	Immediate needle removal
	2g) Needle type (diameter, length, and manufacturer or material)	0.5 × 50 or 80 mm, 0.75 × 50 or 80 mm Dongbang knife acupuncture (Dongbang, South Korea, stainless steel) or<br>0.7 × 50 or 80 mm Ahn’s Small Round Needle (Hansung, South Korea, stainless steel)
**3. Treatment regimen**	3a) Number of treatment sessions	1) Epidural steroid injection single-treatment group (usual care): 0 times<br>2) Epidural steroid injection + deeply inserted acupotomy to

		EX-B2 acupoints treatment group: 8 times
	3b) Frequency and duration of treatment sessions	2 sessions/week for 4 weeks (8 sessions)
**4. Other components of treatment**	4a) Details of other interventions administered to the acupuncture group	Epidural steroid injection
	4b) Setting and context of treatment, including instructions to practitioners, and information and explanations to patients	A practitioner can have a conversation and consultation with the patient if necessary
**5. Practitioner background**	5) Description of participating acupuncturists (qualification or professional affiliation, years in acupuncture practice, other relevant experience)	Professionals with clinical experience of at least 10 years after obtaining a Korean medicine doctor license
**6. Control or comparator interventions**	6a) Rationale for the control or comparator in the context of the research question, with sources that justify this choice	1) Patients with lumbar disc herniation or lumbar spinal stenosis preferentially choose epidural steroid injection as conservative treatment<br>2) There are no relevant studies in the literature because this clinical trial compared a single-treatment group with usual care and a combined treatment group with usual care and acupotomy

	6b) Precise description of the control or comparator. If sham acupuncture or any other type of acupuncture-like control is used, provide details for Items 1 to 3 above	The experimental and control groups were treated with epidural steroid injection (usual care). No additional acupotomy treatment was administered to the control group

Table S2 Outcomes observation schedule

Outcomes	Screening (baseline)	Treatment										Post-treatment
	Week 0		Week 1			Week 2		Week 3			Week 4	Week 8
	Visit 1	Visit 2	Visit 3	Visit 4	Visit 5	Visit 6	Visit 7	Visit 8	Visit 9	Visit 10	Visit 11	Visit 12
Primary outcome												
ODI	√					√					√	√
Secondary outcomes												
NRS	√					√					√	√
EQ-5D	√					√					√	√
MPQ[a]	√					√					√	√
RMDQ	√					√					√	√
Exploratory effectiveness evaluation												
Additional procedures evaluation												√
Early termination		√	√	√	√	√	√	√	√	√	√	√
Rescue medication use		√	√	√	√	√	√	√	√	√	√	√

Treatment responder/non-responder assessment											√	
Safety outcomes												
Adverse events		√	√	√	√	√	√	√	√	√	√	√
PRO-CTCAE						√					√	√
Cost-effectiveness evaluation												
Cost evaluation	√					√					√	√

[a]The MPQ was based on the following: MPQ (sense), MPQ (emotion), MPQ total, MPQ (visual analog scale [VAS]), and MPQ (present pain intensity). The MPQ (sense) was evaluated by questions 1 to 11, while the MPQ (emotion) was evaluated by questions 12 to 15 in the MPQ questionnaire. The MPQ total was calculated as the sum of the MPQ (sense) and MPQ (emotion). The MPQ (VAS) was used to determine the degree of pain on a 10-point scale, while pain intensity was evaluated by the MPQ (present pain intensity) on a 5-point scale.

Abbreviations: EQ-5D, European Quality of Life 5 Dimensions; MPQ, McGill Pain Questionnaire; NRS, Numeral Rating Scale; ODI, Oswestry Disability Index; PRO-CTCAE, Patient-Reported Outcomes version of the Common Terminology Criteria for Adverse Events; RMDQ, Roland–Morris Disability Questionnaire

Table S3 Comparison of the number of additional treatments and the number of responders/nonresponders in the experimental and control groups

		ESI single-treatment group	DAH + ESI treatment group	p-value
Number of additional treatments (excluding three frequencies missing)				
Yes		2 (8.33)	2 (8.70)	1.0000[a]
No		22 (91.67)	21 (91.30)	
Number of responders and nonresponders (ODI)				
Number of ODI	≥ 10%	13 (52)	20 (80)	0.0366[b,c]
	< 10%	12 (48)	5 (20)	
Number of responders and nonresponders (NRS)				
Number of NRS (back pain)	≥ 2.5	8 (32)	13 (52)	0.1520[b]
	< 2.5	17 (68)	12 (48)	
Number of NRS (lower limb pain)	≥ 2.5	9 (36)	15 (60)	0.0894[b]
	< 2.5	16 (64)	10 (40)	

All values are presented as numbers (%).

[a]Fisher's exact test; [b]Chi-square test; [c]$p < 0.05$

Abbreviations: DAH, deeply inserted acupotomy to Hyeopcheok acupoints; ESI, epidural steroid injection; NRS, Numeral Rating Scale; ODI, Oswestry Disability Index.

7

Table S4 Comparison of rescue medication usage between the experimental and control groups

	ESI single-treatment group (n = 20)	DAH + ESI treatment group (n = 21)	p-value
Rescue medication use	297.5 ± 153.5	218.1 ± 118.0	0.0718[a]

All values are presented as the mean ± standard deviation.

[a]Independent-samples t-test

Abbreviations: DAH, deeply inserted acupotomy to Hyeopcheok acupoints; ESI, epidural steroid injection.

Table S5 Comparison of high grade (score ≥ 4) PRO-CTCAE between the experimental and control groups

	ESI single-treatment group	DAH + ESI treatment group	p-value[a]
Visit 3			
Frequency	15 (60)	8 (33.33)	0.0615[b]
Severity	9 (36)	7 (29.17)	0.6101[b]
Interference	6 (24)	3 (12.5)	0.4635[c]
Visit 5			
Frequency	13 (54.17)	7 (30.43)	0.1000[b]
Severity	10 (41.67)	5 (21.74)	0.1429[b]
Interference	5 (20.83)	3 (13.04)	0.7008[c]
Visit 6			
Frequency	12 (50)	9 (39.13)	0.4537[b]
Severity	6 (25)	3 (13.04)	0.4614[c]
Interference	3 (12.5)	2 (8.7)	1.0000[c]

All values are presented as number (%).

[a]When a comparison between the groups was required due to clinically significant changes of grade 4 or higher, generalized estimating equation analysis was used to detect any significant differences.

[b]Chi-square test, [c]Fisher's exact test

Abbreviations: DAH, deeply inserted acupotomy to Hyeopcheok acupoints; ESI, epidural steroid injection; PRO-CTCAE, Patient-Reported Outcomes version of the Common Terminology Criteria for Adverse Events.

Table S6 Comparison of the clinical laboratory examination results between the experimental and control groups

	ESI single-treatment group	DAH + ESI treatment group	p-value
WBC	6.5 ± 1.5	7.0 ± 1.9	0.3485[a]
RBC	4.4 ± 0.5	4.4 ± 0.4	0.8492[a]
Hemoglobin	13.5 ± 1.6	13.3 ± 1.4	0.9538[b]
Hematocrit	40.7 ± 4.2	40.6 ± 3.9	0.7067[b]
Platelets	257.0 ± 52.0	239.8 ± 61.7	0.2578[b]
AST	23.5 ± 4.0	28.9 ± 23.1	0.8692[b]
ALT	23.3 ± 8.2	33.4 ± 52.0	0.9460[b]
ALP	70.8 ± 27.0	69.1 ± 22.8	0.8500[b]
Albumin	4.3 ± 0.2	4.2 ± 0.3	0.0754[a]
BUN	17.2 ± 5.5	14.3 ± 2.5	0.0534[b]
Protein	7.1 ± 0.3	7.1 ± 0.4	0.6007[a]
Creatine	0.8 ± 0.2	0.7 ± 0.2	0.3268[b]
Total cholesterol	185.9 ± 35.2	191.4 ± 41.7	0.6177[a]
Fasting glucose	124.3 ± 44.7	137.8 ± 58.9	0.7648[b]
CRP	2.1 ± 3.5	1.6 ± 1.9	0.4644[b]
ESR	15.3 ± 12.0	20.7 ± 22.7	0.7208[b]
Sodium	138.4 ± 1.9	136.7 ± 3.0	0.0375[b,c]
Potassium	4.3 ± 0.4	4.4 ± 0.4	0.2496[a]
Chloride	104.0 ± 2.1	103.4 ± 2.6	0.7323[b]
Prothrombin time	0.9 ± 0.1	0.9 ± 0.1	0.5363[b]
aPTT	35.0 ± 5.0	34.1 ± 2.9	0.5061[b]

All values are presented as the mean ± standard deviation.

[a]Independent-samples t-test, [b]Wilcoxon rank sum test, [c]$p < 0.05$

Abbreviations: ALP, alkaline phosphatase; ALT, alanine aminotransferase; aPTT, activated partial thromboplastin time; AST, aspartate aminotransferase; BUN, blood urea nitrogen; CRP, C-reactive protein;

DAH, deeply inserted acupotomy to Hyeopcheok acupoints; ESI, epidural steroid injection; RBS, red blood cells; WBC, white blood cells.
